# Supraorbital Nerve Stimulation for Facial Pain

**DOI:** 10.1007/s11916-023-01113-6

**Published:** 2023-05-02

**Authors:** Mohamed Amgad Elsayed Elkholy, Alaa Abd-Elsayed, Ahmed M. Raslan

**Affiliations:** 1 Department of Neurosurgery and Spine Surgery, Ain Shams University, Cairo, Egypt; 2 Chronic Pain Medicine, Department of Anesthesiology, University of Wisconsin-Madison, Madison, WI USA; 3 Department of Neurological Surgery, Oregon Health and Science University, Portland, OR USA

**Keywords:** Supraorbital nerve stimulation, Chronic facial pain, Intractable facia pain, Neuromodulation of facial pain, Trigeminal branch stimulation

## Abstract

**Purpose of Review:**

Chronic facial pain is considered one of the conditions that affect quality of daily life of patients significantly and makes them seek medical help. Intractable facial pain with failed trials of medical treatment and other pain management therapies presents a challenge for neurologists, pain specialists, and neurosurgeons. We describe the possibility of proposing peripheral nerve stimulation of the supraorbital nerves to treat patients with medically intractable facial pain. Stimulation of the supraorbital nerves is performed using percutaneously inserted electrodes that are positioned in the epi-fascial plane, traversing the course of the supraorbital nerves. The procedure has two phases starting with a trial by temporary electrodes that are inserted under fluoroscopic guidance and are anchored to the skin. This trial usually lasts for a few days to 2 weeks. If successful, we proceed to the insertion of a permanent electrode that is tunneled under the skin behind the ear toward the infraclavicular region in which we make a pocket for the implantable pulse generator.

**Recent Findings:**

This procedure has been used in multiple patients with promising results which was published in literature. Literature shows that it provides relief of medically intractable pain, without the need for destructive procedures or more central modulation approaches with a preferable safety profile compared to other invasive procedures.

**Summary:**

Supraorbital nerve stimulation is now considered a valid modality of treatment for patients with medically intractable facial pain and can be offered as a reliable alternative for the patients while discussing the proper plan of management.

## Introduction

The facial region mediates several vital functions with specific biological, emotional, and psychological interpretations. Hence, it has precise capability of sensory discrimination and somatosensory functions. Accordingly, it is provided with rich peripheral innervation with large cortical representation on the homunculus. Also, it presents other forms of sensory inputs which are special sense perceptions in the form of taste and smell sensations. This demonstrates the critical importance of this region in human life and social communications. That is why facial pain results in severe distress that affects human daily life in the form of interference with emotional expressions, speaking, eating, and social communications [1].

The orofacial region is prone to various conditions causing pain whether acute or chronic. Studies reported that 19 to 26% of adults experience facial pain one or more times annually, while the prevalence of chronic facial pain reaches 8 to 15% with increasing percentage with aging populations. Most of these patients seek medical help in the form of clinic visits and frequent medication usage which is reflected on the health care system and causes a significant economic burden to the society, with yearly health care costs approximating to $100 billion in the USA alone [2].

Conditions causing medically refractory facial pain are usually referred for neurosurgical intervention. Neurosurgical management of such cases includes surgical, neuroablative, and neuromodulatory interventions. Neuromodulation gives the patient the advantage of its reversible nature, ability of programming and modifications, and superior safety profile. However, other factors including age, nature of pain, and patient acceptance for implanted devices are crucial when considering neuromodulation [3•].

Peripheral nerve stimulation (PNS) is a neuromodulatory procedure that is widely used for various pain syndromes including orofacial pain. The use of PNS has been increasing over the past few years; however, its concept and original application dates back to the 1960s when Wall and Sweet studied new approaches to suppress neuropathic pain where they inserted an electrode into the infraorbital foramen and monitored a decrease in pain perception with electrical stimulation [4].

Percutaneous trigeminal ganglion stimulation or trigeminal branch stimulation is an evolving modality that offers a reliable management approach with low possible morbidities for patients with severe refractory facial pain. It acts as an effective alternative to opioid medications and can be used to reduce the prescribed opioids to help in solving this ongoing health care problem that is reflected on the society with a great burden on people’s lives [5].

## Facial Pain Classification

The diagnostic term “facial pain” includes multiple clinical conditions such as facial pain of musculoskeletal origin, headache, and migraine syndromes. Orofacial pain syndromes include dental pain, sinusitis, or temporomandibular disorders, and cranial neuralgias such as trigeminal neuralgia (TN) [6].

Painful cranial neuropathies and other facial painful conditions are classified by the International Headache Society as shown in Table 1 [7].

**Table 1:** Classification of Orofacial Pain (by the International Headache Society Classification Committee) [7]

1. Trigeminal neuralgia: Whether classical trigeminal neuralgia or painful trigeminal neuropathy (posttraumatic trigeminal pain, post-acute herpes zoster or postherpetic neuralgia, multiple sclerosis or space-occupying lesions) 2. Glossopharyngeal neuralgia 3. Nervus intermedius (facial nerve) neuralgia 4. Occipital neuralgia 5. Optic neuritis 6. Headache due to ischemic ocular motor nerve palsy 7. Tolosa–Hunt syndrome 8. Para-trigeminal oculo-sympathetic (Raeder) syndrome 9. Recurrent painful ophthalmoplegic neuropathy	

Natoli et al. proposed that chronic migraine is estimated to affect 2–4% of the population, leading to a major problem of medication overuse which has a prevalence of 0.7–1.7%, although acute management of episodic migraines and prophylactic treatment show significant effectiveness. However, chronic migraine patients whose pain is more frequent, disabling, and not significantly reduced by medical treatment remain an unresolved health care problem [8].

Recently, Cruccu et al. published an additional TN classification that could be used for practice and research. Their definition of idiopathic, classical, and secondary TN was based on review of clinical and etiologic features of TN. They proposed that TN caused by neurovascular compression (NVC) is the most frequent form; however, approximately 11% of TN patients show an unclear etiology [9].

## Indications

Although many conditions addressed by peripheral nerve stimulation (PNS) could show response to other modalities of neuromodulation such as spinal cord stimulation, some conditions are better managed with peripheral nerve stimulation or peripheral nerve field stimulation (PNfS) as they are relatively simple procedures with low invasiveness. Currently proposed indications for supraorbital nerve stimulation are [10]:

- Posttraumatic neuralgia
- Postsurgical neuropathic pain
- Neuropathic facial pain with V1 or sometimes V2 distribution
- Postherpetic neuralgia
- Complex regional pain syndrome, especially type II

*Cephalgias like:

- Migraine, both chronic and transformed
- Hemicrania continua
- Cluster headaches
- Chronic daily headaches

*Emerging indications:

- Musculoskeletal pain
- Fibromyalgia

## Patient Selection

One of the major principles of neuromodulation for pain is proper patient selection. Candidates for peripheral nerve stimulation should meet the following criteria [11•]:

1. The facial pain is chronic (> 3–6 months) and either severe or moderate to severe in intensity (higher than 5 on a 0–10 numerical rating score (NRS) of pain intensity).
2. The pain follows the anatomic distribution of the supraorbital nerve.
3. Standard treatment with anti-inflammatory, analgesics, antidepressants, and anticonvulsants was tried and failed, either because the medications were inadequately effective or because of intolerable side effects.
4. The patient maintains some sensation in the area of pain.
5. The pain disappears or significantly improves after local anesthetic block of the supraorbital nerve.
6. The patient showed failed or inadequate response to minimally invasive pain management strategies.
7. The patient has no active infection and no bleeding disorders and is able to tolerate brief general anesthesia.

All these criteria are important, but their importance and value for each patient should be considered on a case-by-case basis.

## Patient Assessment and Workup

The cornerstone for a neurosurgeon in assessing a patient with peripheral neuralgia or neuropathic pain is to determine to what extent the pathophysiology is peripheral or generalized and to exclude the presence of evidence of central sensitization. That is because the more the peripheral component of the patient’s pain, the more he would benefit from peripheral intervention, which is usually less invasive and cheaper and has the prospect of long-term benefit [12].

Patient assessment includes history taking, clinical examination, and investigations to formulate a proper diagnosis describing also the etiology and pathophysiology of the patient’s pain syndrome. The history must be detailed expressing the whole story of the patient condition to detect the possible etiology and pathophysiology, associated symptom complexes of which pain may or may not be the main problem, functional or disability assessment, progression or regression of symptoms, response to prior treatments, and medical history including diabetes [13].

If there is a history of trauma or surgical injury, details of the onset, course, and duration of symptoms and prior treatment records including operation notes will be essential to isolate the presence of neural structural injury. Also, there is a high probability of presence of dysfunction of the nerve whether motor or sensory associated with the pain. There may be a pain-free period before the occurrence or recurrence of symptoms. The distinction between nociceptive, neuropathic, or mixed components of pain on history is crucial in the trauma or postoperative situation, as the treatment will vary according to each setting [14].

Physical examination of the patient with neuropathic pain must not focus only on the neurologic deficit but also consider detecting other neurologic diagnoses that could account for the pain [15]. Examination should also detect other signs beyond the distribution of the peripheral nerve in question, e.g., allodynia [16].

Quantitative sensory testing is used to document sensory and pain thresholds and may propose the pathophysiologic mechanisms, e.g., peripheral or central. They can also be repeated as well as neurologic examinations to detect change or response to therapy [17]. Trigeminal somatosensory evoked potential recording using near-nerve needle stimulation of the Ab afferents of the trigeminal nerve main divisions is advised to be done to all patients with facial pain especially if the symptomatology is not clear. It shows high significance in detecting large fiber pathology in neuropathic facial pain conditions such as cases of classical TN patients with no neurological deficit. But it has rarely been used to study orofacial pain [18].

MRI is a clearly beneficial modality for patients with neuralgia and neuropathies with greater resolution for visualizing the neural structure using multiplanar reconstruction and showing relationship to adjacent structures. Also, it is useful in the detection of concomitant pathologies that may cause facial pain [19].

Psychological screening is recommended to identify any psychosocial problems that may adversely impact the therapy. This screening includes cognitive impairment, substance abuse, and untreated anxiety or depression. It is also done to exclude any unrealistic expectations related to the stimulator [20]. The critical factor before the procedure is proper assessment of opioid medication utilization that has a well-recognized risk profile when used for a long period for treatment of chronic pain and has a clear impact on the post-procedure pain assessment with a well-recognized probability of secondary gains related to substance use disorders [21].

## Mechanism of Neurostimulation

Our perspective of the ability of neurostimulation to alleviate pain has been evolving since Sweet and Wall explained the principles of gate theory to describe their success with PNS. The general principle is thought to involve inhibition and activation of pain-related neural circuitry as well as modulatory pathways of the autonomic system. It has been shown not to work simply by direct electrical stimulation signal cascade but rather include modulating interactions with multiple neurotransmitters, such as g-amino butyric acid (GABA) and adenosine [22].

Although the original mechanism using gate theory to explain pain relief suggested the necessity of the presence of paresthesia for pain coverage and analgesia induction, studies demonstrated that other modes of stimulation such as high frequency stimulation (10 kHz) and burst stimulation can provide at least similar if not better pain relief without the presence of paresthesia. These trials exhibit the idea that we still not fully understand the mechanistic underpinnings of electrical stimulation for pain [23].

Craniofacial stimulation is one of the most successful indications for PNS because of the inability to use spinal cord stimulation or dorsal root ganglion stimulation to treat pain in craniofacial distributions [24].

Peripheral nerve field stimulation, also known as subcutaneous neurostimulation or sometimes targeted subcutaneous stimulation, is an alternative approach in which the surgeon inserts one or more electrodes into the region of most severe pain near the distal branches of the targeted nerves within the subcutaneous tissue. Its stimulation produces paresthesia-mediated pain relief that is diffuse along the painful area but could not be well defined or correlated to a specific dermatome [25].

## Description of the Procedure

### Trial Stage

- The trial stage includes placement of leads along the supraorbital nerve, externalized, and the patient tests the stimulation for a time period ranging from 7 to 14 days. The surgical technique for the trial and permanent leads is similar, but while trial leads are usually sutured to the skin at the site of their exit, permanent leads are sutured to the fascia.
- Mostly performed with conscious sedation that permits for intraoperative testing of the positioned leads
- The patient is positioned with the head in a horseshoe-shaped head holder.
- Exposure of the entire neuropathic area to allow direct access
- Use of fluoroscopy to confirm lead position
- Perioperative antibiotics
- Standard surgical preparation and draping are performed along the entire planned path of the leads visible in the field.

### Surgical Technique

As shown in Fig. 1

- Step 1: The skin is infiltrated with local anesthetic at the entry point, and then a small entry stab incision is made in the lateral forehead (approximately 1.5 cm superolateral to the tip of eyebrow).
- Step 2: A Tuohy needle is advanced in the subcutaneous space overlying the nerve with a trajectory parallel to the nerve or at an angle to it.
- Step 3: The inner stylet of the Tuohy needle is withdrawn, and a guidewire is advanced guided by fluoroscopic imaging.
- Step 4: The Tuohy needle is withdrawn, and a plastic cannula is advanced over the guidewire.
- Step 5: The guidewire is withdrawn.
- Step 6: The trial lead is threaded through the cannula and its position is confirmed. The cannula is withdrawn.
- Step 7: The lead is connected to a temporary testing cable. The patient’s sedation is lightened to enable testing. The patient reports the paresthesias perceived. The optimal coverage can be modulated by changing the combination of anode and cathode contacts and changing amplitude, frequency, and pulse width. If modifications do not result in optimal coverage of the target area, then the leads can be repositioned.
- Step 8: Once optimal position is confirmed, the leads are sutured to the entry site and then covered with a sterile occlusive dressing. The externalized trial leads are connected to the trial stimulator system.
- Step 9: Further programming of the stimulator is performed postoperatively.
- Step 10: Plain radiographs should be obtained to document final trial lead position and to be used as a guide in the permanent placement of leads in case of successful trials.

**Fig. 1:**
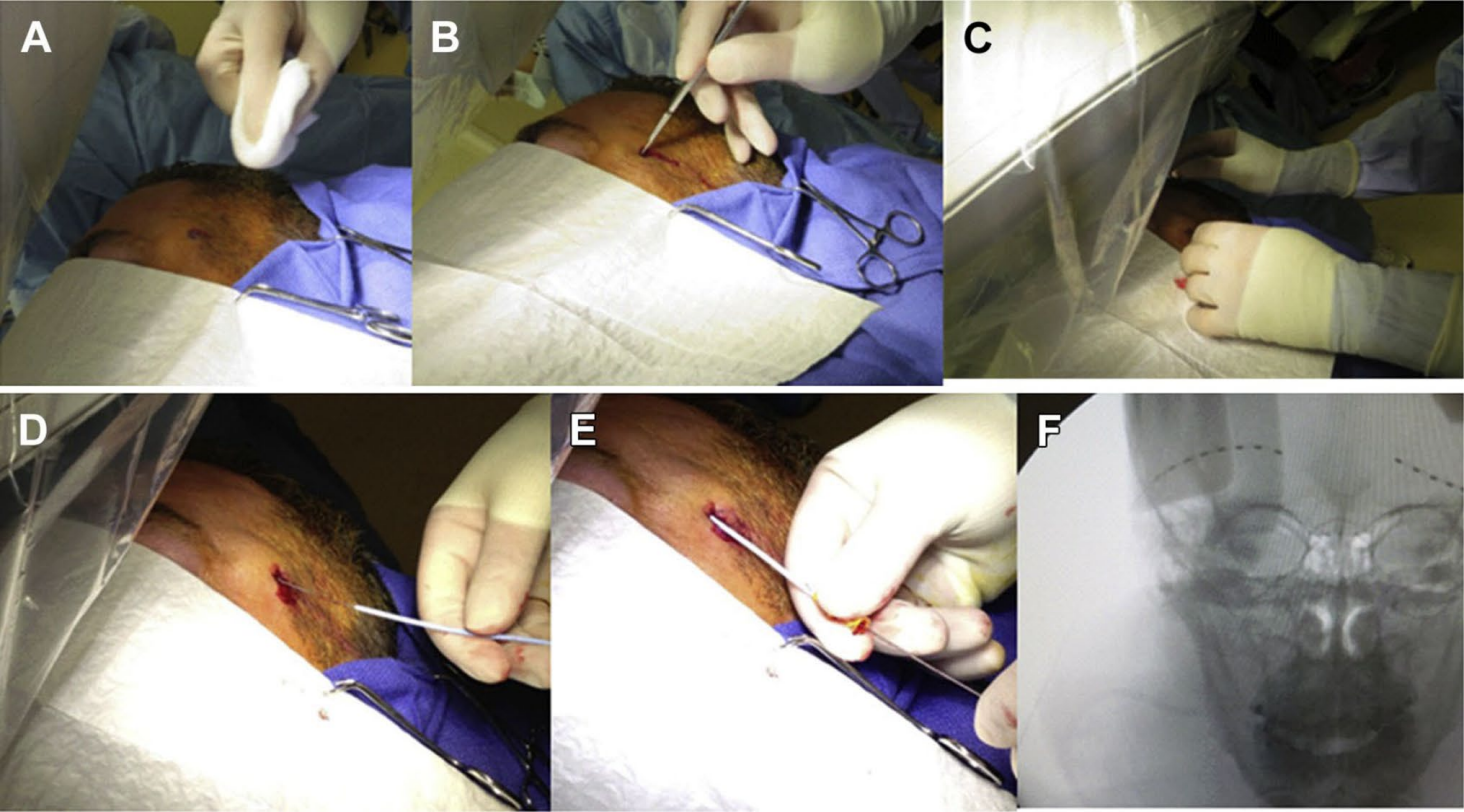
Surgical steps for percutaneous placement of peripheral nerve stimulator of the supraorbital nerve. **A** Skin infiltration with local anesthetic at entry point. **B** Stab incision is done at entry point in the lateral forehead superolateral to the tip of eyebrow. **C** Insertion of Touhy needle guided by fluoroscopy. **D** Advancement of the guide wire, then the plastic cannula is inserted. **E** The lead is introduced toward its position through the cannula guided by fluoroscopy. **F** Fluoroscopic imaging showing the lead in its targeted position [10]

### Post-Trial Phase

- Patients return home the same day of the trial and are asked to continue their daily routine to test the PNS system’s effectiveness. Few adjustments of the stimulator settings could be required to reach optimal relief.
- Patients should keep the externalized cables clean and dry, which may necessitate sponge bathing instead of showering.
- Patients should avoid sudden and vigorous movements that might cause the lead to migrate or dislodge.
- A trial may be considered successful if a patient experiences more than 50% improvement in pain severity. Furthermore, the patient should express satisfaction with the degree of pain relief.
- Patients with a failed trial should not proceed to permanent system implantation.

### Permanent Placement

**Fig. 2:**
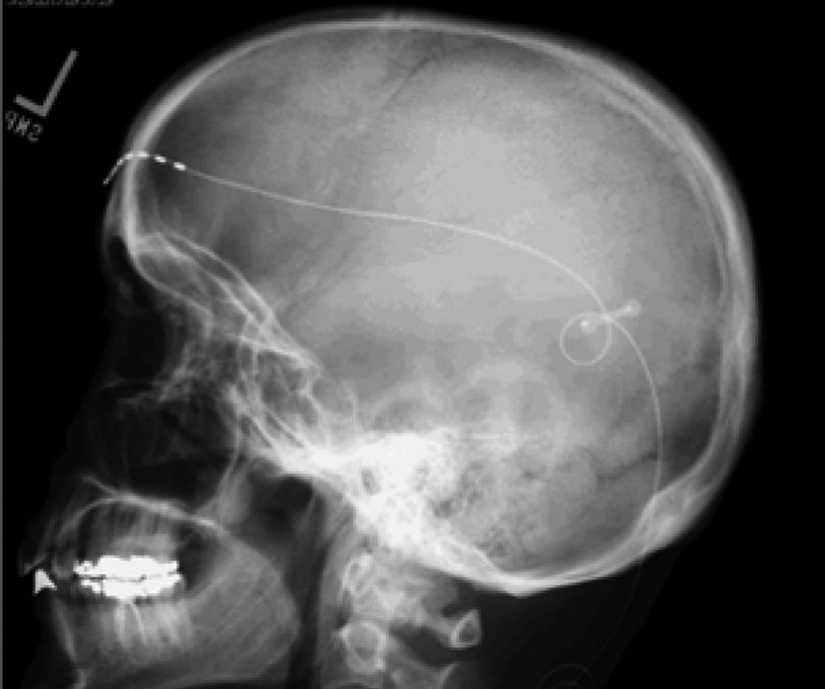
X-ray skull lateral view showing the supraorbital nerve stimulator in the supraorbital region with its anchor and strain-relief loop in the parieto-occipital region [26•]

**Fig. 3:**
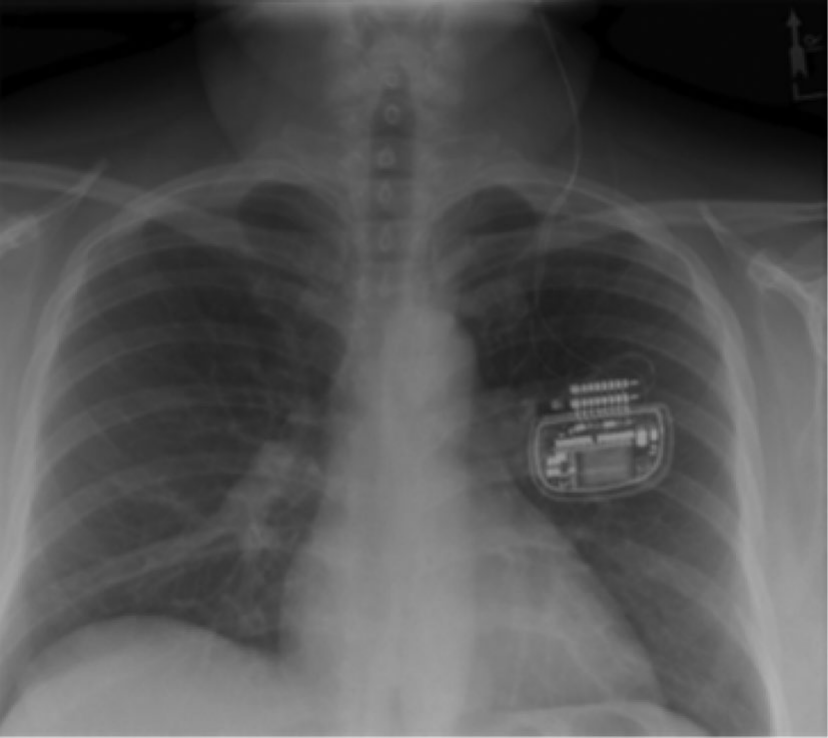
X-ray chest anteroposterior view showing the course of the lead down to the IPG in the generator pocket which is located in the infraclavicular region [10]

- The steps for implantation of the permanent system are similar to those of the trial.
- Most often, it is performed under general anesthesia using fluoroscopy to ensure that lead placement is identical to the trial leads.
- General anesthesia is used also to comfort the patient especially during tunneling of the cables to the generator site.
- Preparation and positioning are similar, with careful attention to accessing the generator site and the ability to tunnel the leads to the generator pocket.

- Step 1: The steps of lead insertion are the same as in the trial procedure.
- Step 2: The leads are anchored to the fascia using a nonabsorbable stitch or with the available anchors with the stimulation system itself.
- Step 3: A strain-relief loop (as shown in Fig. 2) can minimize the risk of migration or fracture due to lead kinking.
- Step 4: Skin incision is done and a subcutaneous pocket is prepared for the implantable pulse generator (IPG). Possible used sites are the flank, abdominal, axillary, subscapular, and infraclavicular areas.
- Step 5: The leads are tunneled from the anchor site to the IPG pocket. Depending on the length of the course, extension cables may be required.
- Step 6: The leads are connected to the IPG (Fig. 3).
- Step 7: Impedance must be checked on the whole system to assess technical integrity, function of the system, and proper alignment of the contact points of the leads to their compatible points at the IPG.
- Step 8: Antibiotic irrigation is performed at the incision sites. The IPG pocket and tunneling site and then the incisions are closed and dressed.

### Post-Permanent Placement

For most patients, the benefit of nerve stimulation is immediate, and they may be able to wean off their opioid medications with satisfactory pain relief. Gradual tapering of medications coordinated with the patient’s prescribing physicians is recommended whether the prescribing physician was the pain medicine specialist or the primary care physician.

Periodic fine adjustments (“tune-ups”) of the stimulation settings may be needed for some patients for better pain coverage and to eliminate extraneous paresthesias especially if the patient notices a change in the quality of stimulation or in the degree of pain relief [10].

If the adjustments failed to gain an adequate pain relief, interrogation of the system should be performed. If there are no electrical faults, then further adjustment of the parameters may regain adequate function or another strategy is used which is a “holiday” period in which the stimulator is turned off. This plan can lead to a resumption of effective stimulation when the device is reactivated [10].

Slavin and Wess demonstrated the efficacy of trigeminal branch stimulation in patients with intractable facial neuropathic pain. In their study, they included 8 patients with insertion of SONS in 5 of them. This stimulation showed an average of 74% pain reduction in the last follow-up [27].

Hann and Sharan studied the efficacy of dual occipital and supraorbital nerve stimulation in chronic migraine patients. They included 14 patients and used SONS for frontal migraines. In this study, SONS showed a significant improvement of frontal migraine that ranged from 70 to 90% as compared to the preprocedural pain scores [26•].

## Adverse Events

The most common complications include lead migration, lead site allodynia, IPG site pain, lead fracture, skin erosion and lead exposure, infection, and neural injury which is relatively rare. Patients should be counseled on these risks and educated about the warning signs that require them to seek immediate assistance.

Hann and Sharan mentioned in their study about supraorbital and occipital nerve stimulation for chronic migraine that the percentage of complications among the 14 patients included in the study was 42.8% for lead migration, 21.4% for lead site pain, and 14.2% for hardware exposure and infection. Supraorbital nerve leads needed revision due to migration in 6 patients which was detected via skull X-ray. An important note drawn from this study was the attempt done by the senior author to avoid lead migration in revision and some later surgeries by creating 2 loops rather than one to reduce the tension on leads and inserting the tip of the stimulator subperiosteally. Also, he used a dog bone-shaped titanium plate to anchor the base of the lead to the skull. This technique was used in 7 patients, and only one of them showed lead migration [26•].
